# Large-Bore Mechanical Thrombectomy With Bivalirudin for Post-CABG HIT-Associated Intermediate- to High-Risk Pulmonary Embolism

**DOI:** 10.1016/j.jaccas.2026.107842

**Published:** 2026-04-10

**Authors:** Roger Lin, Darius G. Aliabadi

**Affiliations:** a Internal Medicine Graduate Medical Education, Southeast Health, Dothan, Alabama, USA; b Cardiovascular Institute, Southeast Health, Dothan, Alabama, USA

**Keywords:** anticoagulation, postoperative, pulmonary circulation, right-sided catheterization, systolic heart failure, thrombosis, thrombus

## Abstract

**Background:**

Pulmonary embolism (PE) after coronary artery bypass grafting (CABG) is a rare but high-risk event. Management is challenging when PE occurs with heparin-induced thrombocytopenia (HIT), limiting thrombolytic and heparin-based strategies.

**Case Summary:**

A 59-year-old man developed hypoxemic respiratory failure within 2 weeks after CABG. Computed tomography angiography demonstrated bilateral main and lobar PEs with right-heart strain. HIT was confirmed by studies. Given recent surgery and active HIT, thrombolysis and heparin were contraindicated. He underwent urgent bilateral large-bore mechanical thrombectomy with intraprocedural bivalirudin, resulting in clot reduction and respiratory failure resolution.

**Discussion:**

This case demonstrates the feasibility of large-bore mechanical thrombectomy with bivalirudin for post-CABG intermediate- to high-risk PE with HIT. Published experience with exclusive bivalirudin during large-bore pulmonary thrombectomy remains limited, and this case adds to the evidence for postsurgical PE management.

**Take-Home Message:**

Large-bore mechanical thrombectomy may provide effective reperfusion when thrombolysis is contraindicated, with bivalirudin serving as a nonheparin anticoagulant in active HIT.

## Visual Summary

**Figure:**
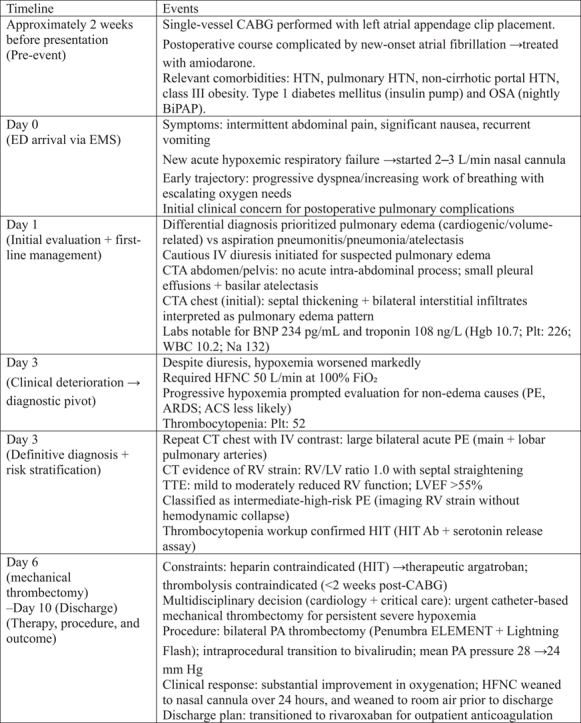

## History of Presentation

A 59-year-old man arrived via emergency medical services with intermittent abdominal pain, significant nausea, and recurrent vomiting. On arrival, he had acute hypoxemic respiratory failure, requiring 2 to 3 L/min nasal cannula. During his hospitalization, his respiratory status progressively worsened, with escalating oxygen needs. Eventually, he required high-flow nasal cannula support with 100% Fio_2_ at 50 L/min.

**Take-Home Message**

• Large-bore mechanical thrombectomy with bivalirudin anticoagulation may offer effective reperfusion for post-CABG intermediate- to high-risk PE with HIT when thrombolysis and heparin are contraindicated.

## Past Medical History

The patient had a history of coronary artery disease and had recently (<2 weeks before presentation) undergone single-vessel coronary artery bypass grafting (CABG), during which a left atrial appendage clip was placed. His postoperative recovery was complicated by new-onset atrial fibrillation, which was treated with amiodarone. Comorbidities included class III obesity, hypertension, pulmonary hypertension, noncirrhotic portal hypertension, type 1 diabetes mellitus managed with an insulin pump, and obstructive sleep apnea requiring nocturnal BiPAP.

## Differential Diagnosis

Initial concerns included postoperative pulmonary edema (cardiogenic or volume-related), aspiration pneumonitis, pneumonia, and atelectasis. Given the patient's progressive hypoxemia despite administration of diuretics, alternative causes were considered. These included pulmonary embolism (PE), acute respiratory distress syndrome, and, less likely, acute coronary syndrome.

## Investigations

**Figure 1:**
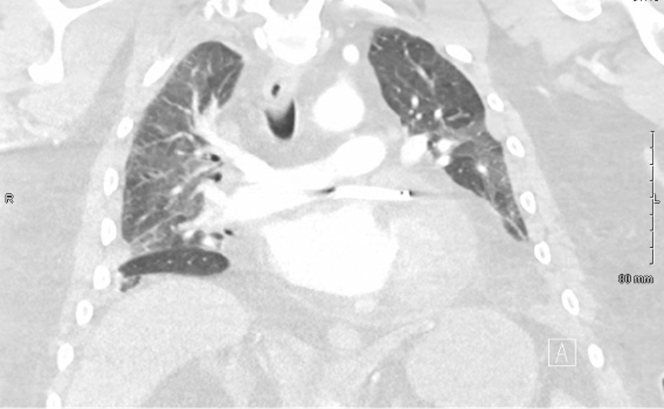
Chest Computed Tomography Angiography Demonstrating No Pulmonary Embolism Coronal contrast-enhanced imaging shows no filling defect to the level of the proximal segmental pulmonary arteries. Small bilateral pleural effusions are present with associated lower lobe subsegmental atelectasis. No pulmonary mass or pneumothorax is identified.

The initial computed tomography angiography (CTA) of the abdomen and pelvis showed unremarkable findings, except for small pleural effusions and basilar atelectasis. CTA of the chest indicated interlobular septal thickening and bilateral interstitial infiltrates, which are consistent with pulmonary edema (Figure 1). Laboratory evaluations revealed a hemoglobin level of 10.7 g/dL, a platelet count of 226 × 10^9^/L, a white blood cell count of 10.2 × 10^9^/L, sodium levels at 132 mmol/L, a B-type natriuretic peptide level of 234 pg/mL, and a troponin level of 108 ng/L.

**Figure 2:**
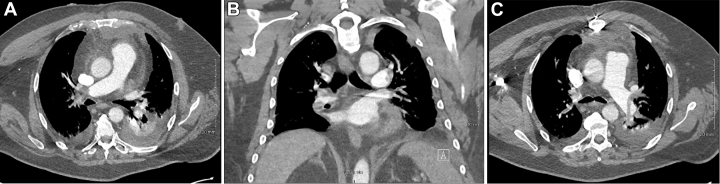
Chest Computed Tomography Angiography Demonstrating Bilateral Pulmonary Emboli (A) Contrast-enhanced imaging shows a linear intraluminal filling defect within several right upper lobe segmental and subsegmental pulmonary arteries. (B) A large filling defect is present in the right interlobar pulmonary artery with extension into the right middle and lower lobe branches. (C) Axial contrast-enhanced imaging demonstrates a linear intralinal filling defect within the left interlobar pulmonary artery extending into the left upper and lower lobe segmental branches.

**Figure 3:**
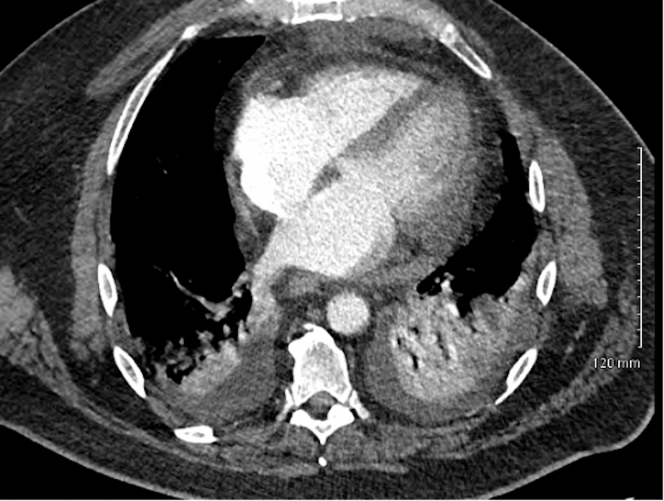
Chest Computed Tomography Angiography Demonstrating Right Ventricular Enlargement Axial contrast-enhanced imaging shows a right ventricular–to–left ventricular diameter ratio of 1.0 with straightening of the interventricular septum.

Because of worsening hypoxemia, a repeat chest computed tomography with intravenous contrast was performed, which revealed large bilateral acute PEs involving the main and lobar pulmonary arteries (Figures 2A and 2B). Additionally, there was bibasilar multisegmental atelectasis and small pleural effusions. The right ventricle–to–left ventricle (RV/LV) ratio was 1.0 (Figure 3), with straightening of the interventricular septum, raising concerns for right-sided heart strain. Transthoracic echocardiography demonstrated mild to moderately reduced RV systolic function and an LV ejection fraction of >55%. The patient's presentation was consistent with intermediate- to high-risk PE. Further evaluation for thrombocytopenia confirmed heparin-induced thrombocytopenia (HIT), with a platelet count of 52 × 10^9^/L, positive HIT antibody testing, and a positive serotonin release assay. The timing and magnitude of thrombocytopenia were consistent with typical-onset HIT after recent heparin exposure during CABG.

## Management (Medical/Interventions)

The patient was initially treated with cautious intravenous diuretics for suspected pulmonary edema and was started on nonheparin anticoagulation with therapeutic argatroban for HIT. Despite these measures, his respiratory failure progressed. A multidisciplinary team determined that systemic thrombolysis posed a prohibitive bleeding risk given the patient's recent CABG (<2 weeks), and heparin was contraindicated owing to HIT.

Given persistent severe hypoxemia and the contraindications to both thrombolysis and heparin, the patient underwent urgent bilateral pulmonary artery mechanical thrombectomy. Under moderate conscious sedation, ultrasound-guided right femoral venous access was obtained, and a 9-F sheath was placed. The patient was on argatroban, with an activated clotting time of 243 seconds; during the procedure, anticoagulation was switched to bivalirudin.

Hemodynamic assessment with a Swan-Ganz catheter revealed sinus tachycardia at 120 beats/min, a Fick cardiac output of 7.7 L/min (cardiac index: 3.0 L/min/m^2^), and a mean pulmonary arterial pressure of 28 mm Hg. Pulmonary angiography showed large mobile thrombi in the right and left main pulmonary arteries, extending into the lobar branches (Video 1). The sheath was upsized to a 17-F Penumbra Element system, and aspiration thrombectomy was performed using the Penumbra Lightning Flash system, with multiple passes in both pulmonary arteries. Large volumes of fresh thrombus were removed, with an estimated blood loss of 300 mL.

**Figure 4:**
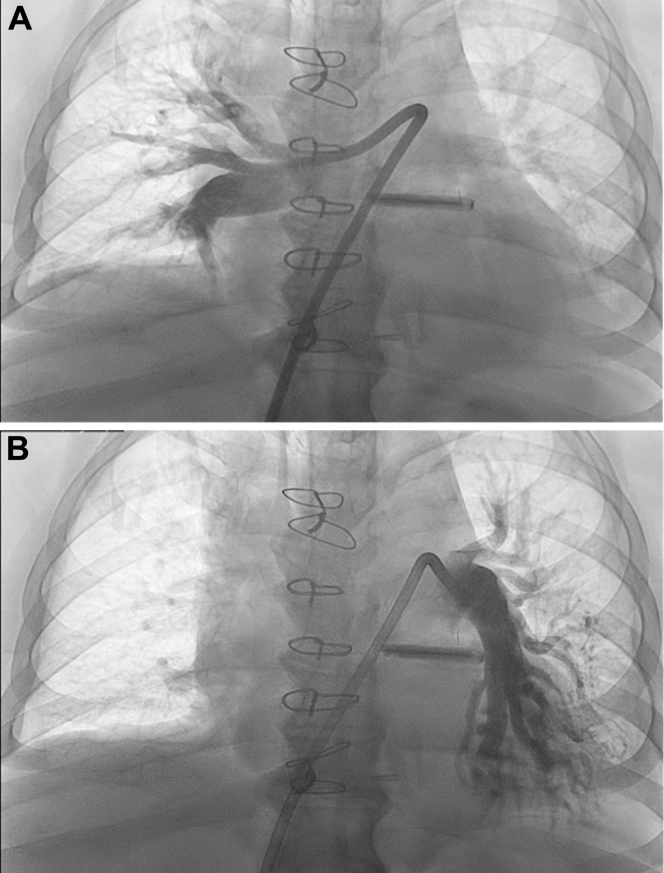
Right and Left Pulmonary Artery Angiography After Mechanical Thrombectomy Postaspiration angiography demonstrates a substantial reduction in thrombus burden within the (A) right pulmonary arterial system and (B) left pulmonary arterial system, with improved distal perfusion.

Final angiography demonstrated a marked reduction in clot burden and improved pulmonary perfusion (Figures 4A and 4B), with mean pulmonary arterial pressure decreasing from 28 to 24 mm Hg. The procedure was completed without complications after hemostasis was achieved.

## Outcome and Follow-Up

After thrombectomy, oxygenation improved substantially, and high-flow nasal cannula support was successfully weaned over the next 24 hours. The patient remained clinically stable on argatroban and was transitioned to rivaroxaban for long-term anticoagulation before discharge planning.

## Discussion

This case illustrates the management challenges of intermediate- to high-risk PE occurring shortly after CABG in the setting of confirmed HIT and rapidly progressive hypoxemic respiratory failure. HIT is a prothrombotic immune-mediated reaction driven by antibodies to platelet factor 4–heparin. HIT occurs in approximately 1% to 2% of patients after cardiac surgery and carries substantial thrombotic risk if untreated.^1^ Guideline-based management requires immediate discontinuation of all heparin products and initiation of a nonheparin anticoagulant (eg, argatroban or bivalirudin).^2^

Risk stratification guided escalation planning. CTA demonstrated right-heart strain (RV/LV ratio of 1.0 with interventricular septal straightening), and transthoracic echocardiography showed mild to moderately reduced RV systolic dysfunction, supporting classification as intermediate-risk PE. With RV dysfunction, elevated troponin, and increasing oxygen requirement, this presentation aligns with intermediate-high risk.

In intermediate- to high-risk PE, systemic anticoagulation with close monitoring is standard, while reperfusion therapy is reserved for patients who deteriorate or have concerning physiology despite anticoagulation.^3^ The PEITHO trial demonstrated that systemic thrombolysis with tenecteplase reduced hemodynamic decompensation by 3 percentage points but increased major bleeding by 9 percentage points and hemorrhagic stroke by 2 percentage points, leading to recommendations against routine systemic thrombolysis in this population.^4^ In the present case, recent cardiothoracic surgery (CABG <2 weeks prior) posed significant bleeding risk, making systemic thrombolysis unfavorable. Current guidelines list major surgery within 3 weeks as a relative contraindication to fibrinolytic therapy, shifting the risk-benefit balance toward catheter-based reperfusion.^5^

Contemporary randomized data support mechanical thrombectomy in selected patients when rapid physiologic improvement is desired and thrombolysis carries high risk. In the PEERLESS trial, large-bore mechanical thrombectomy outperformed catheter-directed thrombolysis on a hierarchical composite endpoint, driven by fewer episodes of clinical deterioration/bailout therapy (1.8% vs 5.4%, *P* = 0.04) and reduced implantable cardioverter-defibrillator use (41.6% vs 98.6% admissions), without differences in mortality or major bleeding.^6^ In the STORM-PE trial, computer-assisted vacuum thrombectomy plus anticoagulation produced greater reduction in RV/LV ratio at 48 hours (0.52 ± 0.37 vs 0.24 ± 0.40; difference, 0.27; 95% CI: 0.12-0.43; *P* < 0.001) compared with anticoagulation alone, with comparable major adverse event rates (4.3% vs 7.5%, *P* = 0.681), supporting thrombectomy for early improvement in intermediate- to high-risk PE.^7^

The Penumbra aspiration system was selected in the setting of class III obesity, multifocal lobar and segmental clot burden, and escalating hypoxemia with concern for hemodynamic compromise. Its continuous aspiration mechanism is well suited for distal and segmental thrombus extraction, allowing more effective engagement of smaller caliber branches compared with larger-bore platforms designed for central clot retrieval. In addition, its smaller sheath profile may facilitate femoral venous access and potentially reduce access site–related complications, while rapid deployment enabled prompt reperfusion and RV unloading. In this time-sensitive clinical deterioration, operator familiarity and procedural expediency prioritized expedited clot extraction despite the potential for increased blood loss inherent to continuous aspiration systems.

A key procedural consideration was anticoagulation selection in the setting of HIT. Argatroban and bivalirudin are the primary nonheparin anticoagulants in the United States. Argatroban was transitioned to bivalirudin for thrombectomy given its established use in catheter-based interventions, rapid onset and shorter half-life, and more predictable intraprocedural anticoagulation with activated clotting time monitoring, permitting controlled offset at procedure completion. Although bivalirudin is approved by the U.S. Food & Drug Administration for percutaneous coronary intervention in patients with HIT or prior HIT, published experience with exclusive bivalirudin anticoagulation during large-bore pulmonary thrombectomy remains limited. Cardiovascular data demonstrate comparable ischemic outcomes and reduced bleeding versus heparin in over 19,000 patients.^8^ In a retrospective series of 461 HIT patients treated with bivalirudin, new thrombosis occurred in 4.6% and major bleeding in 7.6%, without HIT-related amputations.^9^ The present case supports the feasibility of bivalirudin during aspiration thrombectomy when both heparin and thrombolysis are undesirable, underscoring the importance of multidisciplinary, physiology-driven decision-making.

After stabilization and platelet recovery, direct oral anticoagulants (DOACs) have increasingly been used for HIT-associated thrombosis. The 2018 American Society of Hematology guidelines conditionally recommend DOACs, with rivaroxaban having the most published experience.^2^ In a systematic review of 275 patients treated with DOACs for acute HIT, thrombosis rates ranged from 0% to 8.3%, and major bleeding occurred in 1 patient.^10^ Transition from parenteral therapy can occur without overlap once clinically stable. Overall, this case supports aspiration thrombectomy under bivalirudin anticoagulation as a feasible strategy for complex post-CABG intermediate- to high-risk PE with HIT when thrombolysis and heparin are contraindicated.

## Conclusions

Intermediate- to high-risk PE shortly after CABG, complicated by confirmed HIT and rapidly escalating hypoxemic respiratory failure, presents a therapeutic dilemma in which thrombolysis and heparin-based anticoagulation may be contraindicated. This case demonstrates that large-bore catheter-directed mechanical thrombectomy can achieve effective reperfusion and rapid clinical improvement when deterioration persists despite nonheparin anticoagulation. The successful use of bivalirudin for intraprocedural anticoagulation supports its feasibility during aspiration thrombectomy in HIT-associated PE and provides pragmatic guidance for managing similarly complex postoperative patients.

## Funding Support and Author Disclosures

The authors have reported that they have no relationships relevant to the contents of this paper to disclose.

## Appendix

Video 1 Pulmonary Angiography Anteroposterior projection pulmonary angiography performed with injection of 40 mL of contrast at 20 mL/s demonstrates large bilateral pulmonary emboli with extensive filling defects involving the right and left pulmonary arterial systems.
